# CT Perfusion Imaging in Patients with Acute Ischemic Stroke: The Role of Premorbid Statin Treatment

**DOI:** 10.3390/tomography11050054

**Published:** 2025-05-06

**Authors:** Eliseo Picchi, Francesca Di Giuliano, Noemi Pucci, Fabrizio Sallustio, Silvia Minosse, Alfredo Paolo Mascolo, Federico Marrama, Valentina Ferrazzoli, Valerio Da Ros, Marina Diomedi, Massimo Federici, Francesco Garaci

**Affiliations:** 1 Diagnostic Imaging Unit, Department of Biomedicine and Prevention, University of Rome Tor Vergata, Via Montpellier 1, 00133 Rome, Italyvalerio.da.ros@uniroma2.it (V.D.R.); francesco.garaci@uniroma2.it (F.G.); 2 Neuroradiology Unit, Department of Biomedicine and Prevention, University of Rome Tor Vergata, Via Montpellier 1, 00133 Rome, Italy; francesca.di.giuliano@uniroma2.it (F.D.G.);; 3 Comprehensive Stroke Center, Department of Systems Medicine, University of Rome Tor Vergata, Via Montpellier 1, 00133 Rome, Italymarina.diomedi@uniroma2.it (M.D.); 4 Department of Systems Medicine, University of Rome Tor Vergata, Via Montpellier 1, 00133 Rome, Italy; federicm@uniroma2.it

**Keywords:** statin therapy, perfusion CT, acute ischemic stroke, ischemic core, ischemic penumbra, endovascular treatment

## Abstract

Background. Statins appear to be useful in patients with acute ischemic stroke. Our aim was to evaluate the association between premorbid statin treatment and CT perfusion characteristics of acute ischemic stroke. Methods. A retrospective analysis of patients with acute stroke secondary to occlusion of large vessels in the anterior circulation was performed to assess collateral flow, ischemic core volume, and ischemic penumbra using CT angiography and CT perfusion maps. Fisher’s exact test was used to compare baseline characteristics of patients in the two groups. The Wilcoxon rank-sum test for independent groups was used to compare all variables obtained for the two different groups with and without statin use. Results. We identified 61 patients, including 29 treated with statins and 32 not treated with statins before stroke onset matched by age, gender, and vascular risk factors except for hypercholesterolemia. The statin group showed lower National Institutes of health Stroke Scale scores at onset (14 ± 6.1 vs. 16 ± 4.5; *p* = 0.04) and lower volumes of brain tissue characterized by impaired cerebral blood flow (CBF), cerebral blood volume (CBV), and $T_{max}^{9.5-25s}$; otherwise, no statistically significant difference was found in the volume of the $T_{max}^{16-25s}$ between the two groups. Conclusions. Premorbid statin treatment is associated with a favorable imaging condition of acute ischemic stroke in terms of ischemic core and ischemic penumbra volume.

## 1. Introduction

Therapy with 3-hydroxy-3-methylglutaryl coenzyme A reductase inhibitors (statins) has shown beneficial effects in primary and secondary stroke prevention [1,2,3,4]. A systematic review and meta-analysis of observational and randomized trials involving more than 113,000 patients demonstrated that pre-stroke statin use was associated with good functional outcome and reduced likelihood of death at 90 days [5]. Another meta-analysis showed that statin pretreatment in patients with acute ischemic stroke was associated with a reduction in final infarct volume [6].

Although the exact mechanism responsible for those beneficial effects of statin therapy is unknown, it appears to improve endothelial nitric oxide synthase (eNOS) function, vasodilation, and promotion of arteriogenesis [7,8,9,10,11,12]. The combination of those effects results in collateral flow increase, thereby counteracting the reduction in cerebral blood flow, preserving the ischemic penumbra, and delaying complete infarction [13,14,15].

Large vessel occlusions (LVOs) account for approximately 20–40% of acute ischemic strokes [16]. Endovascular therapy (EVT) has become the gold standard treatment for patients with ischemic stroke due to with LVOs [17].

Neuroimaging plays a key role in the diagnosis and management of neurological disorders, providing a precise view of both structural and functional aspects of the brain [18]. In the diagnostic context of acute stroke, computed tomography (CT), CT angiography (CTA) and CT perfusion (CTP) studies are the most suitable imaging techniques, allowing rapid and accurate assessment of blood vessels and cerebral perfusion, which is essential for identifying regions with impaired perfusion and determining the extent of infarction. This information can improve clinical outcomes and reduce the long-term effects of stroke, thanks to the early selection of patients who may benefit from reperfusion therapies such as thrombolysis or mechanical thrombectomy [14,15].

The aim of this original study is to evaluate the association between premorbid statin treatment and CT perfusion characteristics of patients with acute ischemic stroke due to LVO before EVT compared to patients without statins and investigate the putative protective role of statins.

## 2. Materials and Methods

We performed a retrospective analysis of patients with acute ischemic stroke secondary to LVO of the anterior circulation admitted to our emergency department. Inclusion criteria were men or women over 18 years of age with onset of signs and symptoms of anterior ischemic stroke within 6 h due to occlusion of the proximal segment (M1) of the middle cerebral artery (MCA) or tandem occlusion who underwent to multiphasic computed tomography angiography (mCTA) and computed tomography perfusion (CTP) before the administration of EVT.

Exclusion criteria included isolated internal carotid artery or distal MCA occlusions, posterior circulation strokes, and poor image quality.

According to current guidelines, eligible patients received intravenous thrombolysis prior to EVT or underwent EVT directly.

Patients with pre-stroke statin treatment were compared to patients without pre-stroke statin treatment, matched for age, gender, and vascular risk factors except for hypercholesterolemia, which is the main indication for statin treatment. They were also matched for systolic and diastolic blood pressure and glycemia at the time of emergency admission and for the time between symptom onset and imaging given the predictable effect of these variables on neuroimaging findings [19,20,21]. The following variables were compared between groups: admission National Institutes of Health Stroke Scale (NIHSS), collateral flow, global hypoperfused volume (ischemic core + ischemic penumbra), ischemic core volume, and ischemic penumbra volume.

This observational, retrospective study was approved by the local ethical committee (R.S. 25/18) and was conducted in accordance with the ethical standards published in the Declaration of Helsinki of 1975 and its later amendments. Informed consent was obtained from each patient or from the patient’s next of kin if the patient was mentally incapable of giving consent.

### 2.1. Imaging Protocol

The imaging protocol included axial non-contrast CT (NCCT), mCTA, and CTP performed with a 64-slice CT scanner (Lightspeed; General Electric Healthcare, Waukesha, WI, USA).

The NCCT scan was performed with the following parameters: scan type: axial; gantry tilt: orbito-meatal plane; slice thickness: 2.5 mm/5 mm base/cerebrum; interval: 20 mm; kV: 120; mA: 120–300; rotation time: 1 s; matrix: 512 × 512.

The mCTA was acquired during intravenous administration of 50 mL of iodinated contrast media (370 mgI/mL) injected at a rate of 4.5 mL/second, followed by 40 mL of saline bolus at 5 mL/second. mCTA included three phases. The first phase was acquired from the aortic arch to the vertex (scan type: helical full; slice thickness: 0.625 mm, kV: 100; mA: 150–600; rotation time: 0.5 s; acquisition time: seven seconds). The second and third phases were acquired with the same parameters from the skull base to the vertex.

For the CTP protocol, 50 mL of iodinated contrast agent (370 mgI/mL) was power injected at a rate of at least 4.5 mL/second, followed by a 50 mL saline flush at 5 mL/second. The large detector array (8 cm) allowed evaluation of the entire MCA territory. The protocol consisted of two phases (scan type: axial-shuttle; slice thickness: 5 mm; kV: 80; mA: 200; rotation time: 0.5 s). The first phase started with a five-second delay after contrast injection, and 22 acquisitions were repeated on the same 8 cm section (total scan time: 60.6 s). The second phase was delayed by 15 s and consisted of one acquisition on the same section (total scan time: 15 s).

### 2.2. Image Analysis

Collateral flow images were reviewed independently by two investigators (E.P. and F.D.G.) with five years of experience, who were blinded to the treatment arm.

Collaterals were scored on a 0–3 scale derived from the Prolyse in Acute Cerebral Thromboembolism (PROACT) II trial (0: no collaterals; 1: collaterals in the periphery of the ischemic area; 2: collaterals filling 50–100% of the ischemic area; 3: collaterals filling 100% of the ischemic area) [22]. The collateral flow assessment was performed using multiphase CT angiography source images, with experienced investigators evaluating the site of occlusion and grading the extent of collateral filling in the ischemic territory. This assessment was based on the degree of vessel enhancement around the lesion; specifically, the site of vascular occlusion and the area showing a paucity of vessels were recorded using the CTA images with maximum intensity projection (MIP). The total vascular territory supplied by the occluded arterial segment was compared to the area with a paucity of vessels, with the mismatch between these regions representing enhancing vessels interpreted as collaterals. For the purpose of analysis, collateral flow was further categorized as “good” (grades 2 and 3) or “poor” (grades 0 and 1), according to a previously described method [23].

CTP images were processed using a commercially available delay-insensitive deconvolution software (GE Healthcare CT Perfusion 4D, Milwaukee, WI, USA) on the Advantage Workstation 4.7 provided by GE Healthcare. The arterial input function was manually selected from the basilar artery or along the contralateral internal carotid artery using an in-slice region of interest (ROI).

**Figure 1:**
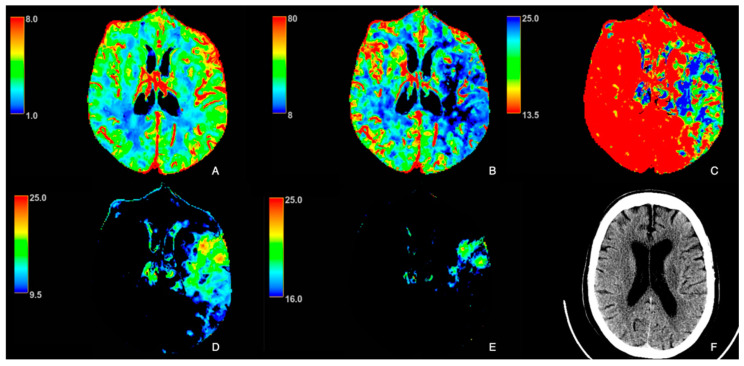
A 83-year-old female patient with acute ischemic stroke due to left middle cerebral artery occlusion under statin therapy. (**A**): Cerebral blood volume (CBV); (**B**): cerebral blood flow (CBF); (**C**): mean transit time (MTT); (**D**): $T_{max}^{9.5-25s}$; (**E**): $T_{max}^{16-25s}$; (**F**): axial CT brain. NIHSS onset: 5.

**Figure 2:**
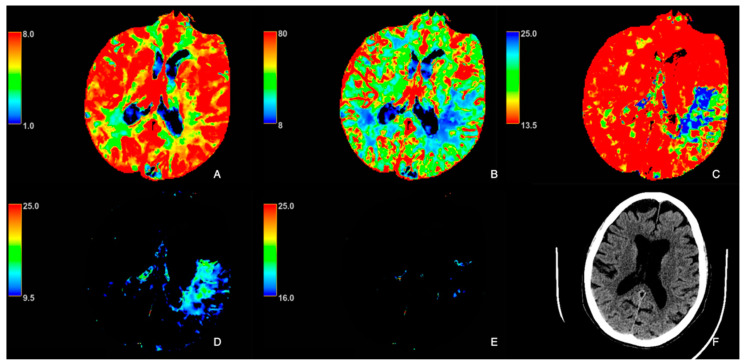
A 74-year-old female patient with acute ischemic stroke due to left middle cerebral artery occlusion. (**A**): Cerebral blood volume (CBV); (**B**): cerebral blood flow (CBF); (**C**): mean transit time (MTT); (**D**): $T_{max}^{9.5-25s}$; (**E**): $T_{max}^{16-25s}$; (**F**): axial CT brain. NIHSS onset: 17.

Maps of cerebral blood flow (CBF) (mL/min/100 g), cerebral blood volume (CBV) (mL/100 g), mean transit time (MTT) (seconds, s), and Tmax (seconds, s) were obtained by deconvolution of tissue time–density curves and the arterial input function using a delay-insensitive algorithm. The Tmax maps were post-processed using two different thresholds: the first, between 16 s and 25 s, was used to obtain the map for the delineation of the ischemic core ($T_{max}^{16-25s}$), while the second, between 9.5 and 25 s, was used to obtain the map for the identification of the hypoperfused area ($T_{max}^{9.5-25s}$) (Figure 1 and Figure 2), as previously reported and according to the literature [24,25,26,27,28,29].

For each patient, an experienced radiologist (FDG), who was blinded to the patient’s clinical outcome, manually outlined the areas with altered perfusion on different perfusion maps to obtain volumes (CBF_v_: volume in CBF map; CBV_v_: volume in CBV map; $T_{max,v}^{16-25s}$: volume in $T_{max}^{16-25s}$ map; $T_{max,v}^{9.5-25s}$: volume in $T_{max}^{9.5-25s}$ map).

### 2.3. Statistical Analysis

Fisher’s exact test was used to compare baseline characteristics of patients in the two groups. Medians and 95% confidence intervals (95% CIs) of the medians of all variables ($T_{max,v}^{16-25s}$, $T_{max,v}^{9.5-25s}$, CBV_v_, CBF_v_, and $T_{max,v}^{9.5-25s}$ − CBF_v_) were calculated for the whole patient population. The Kolmogorov–Smirnov test was used to verify the normal distribution. Because the data were not normally distributed, we assessed the data using a non-parametric statistic. The Wilcoxon rank-sum test for independent groups was used to compare all variables obtained for the two different groups with and without statin use. Box and whisker plots were used to display statistical summaries of the values ($T_{max,v}^{16-25s}$, $T_{max,v}^{9.5-25s}$, CBV_v_, CBF_v_, $T_{max,v}^{9.5-25s}$ − CBF_v_, and NIHSS scores). In each box, the central marker is the median. The edges of the box correspond to the 25th and 75th percentiles, and the horizontal lines extend from the minimum to the maximum value. Outliers are marked with a cross. A *p*-value < 0.05 was considered statistically significant. MATLAB version 9.3.0, release 2017b (MathWorks, Natick, MA, USA) was used for data analysis.

## 3. Results

We retrospectively reviewed the clinical and radiological data of 90 stroke patients admitted to the Stroke Unit of our hospital. We identified 61 patients, of whom 29 were treated with statin and 32 were not statin treated before stroke onset according to the above criteria. The two groups of patients were matched for age, sex, and vascular risk factors except for hypercholesterolemia.

The patients included 11 males and 18 females, with a mean age of about 76 years, in the statin groups as well as 11 males and 21 females, with a mean age of about 73 years, in the non-statin group. We found no statistically significant difference in age (*p* = 0.5, Mann–Whitney U test) and gender (*p* = 0.6 Chi-squared test) between the two groups.

Sample size was assessed as 53 people according to the Z formula and a confidence interval of 95% with 80% power to detect any significant difference between the statin and non-statin groups with a significance level of 0.05.

Although it is difficult to accurately identify patients with ischemic stroke and concomitant statin treatment, we assumed that the annual incidence of stroke patients treated with statin could range between 3% and 5% to calculate the sample size prospectively. After studying the variables prospectively, we calculated the effect size (Cohen’s d) for the variables of interest ($T_{max,v}^{16-25s}$, $T_{max,v}^{9.5-25s}$, CBV_v_, and CBF_v_), resulting in a value greater than 0.08.

Table 1 shows demographics, baseline characteristics, vascular risk factors, and previous pharmacological therapy for the two groups.

No differences were found for atrial fibrillation (*p* = 0.1), systemic hypertension (*p* = 0.2), smoking (*p* = 0.1), diabetes (*p* = 0.1), TIA/ictus (*p* = 0.7), and coronary artery disease (0.1) except for hypercholesterolemia which was, as expected, more common in the statin group (*p* < 0.001).

The median NIHSS score was 13 in the statin group and 17 in the non-statin group, with a significantly lower NIHSS score at symptom onset in the statin group (*p* < 0.001).

The $T_{max,v}^{16-25s},T_{max,v}^{9.5-25s}$, CBV_v_, CBF_v_, and $T_{max,v}^{9.5-25s}$ − CBF_v_ values derived from the two groups are shown in Table 2.

The differences in the $T_{max,v}^{9.5-25s}$, CBV_v_, CBF_v_, and $T_{max,v}^{9.5-25s}$ − CBF_v_ values were statistically significant for both groups.

**Figure 3:**
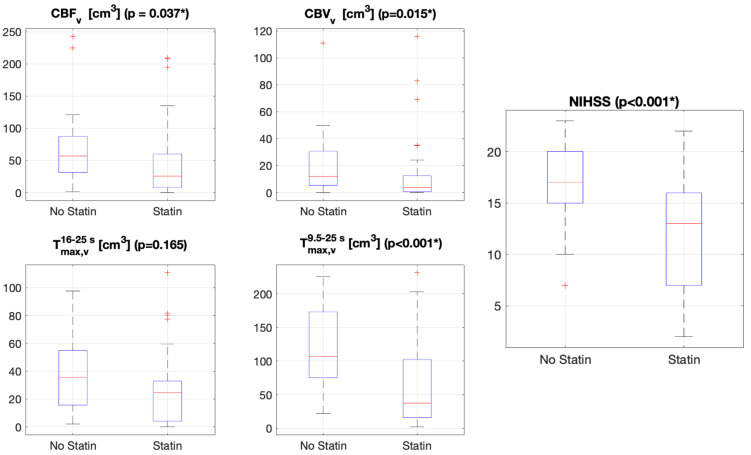
Box and whisker plots of neuroradiological and clinical variables. Abbreviations: CBF_v_, volume in cerebral blood flow (CBF) map; CBV_v_, volume in cerebral blood volume (CBV) map;$\;T_{max,v}^{16-25s}$, volume in $T_{max}^{16-25s}$ map; $T_{max,v}^{9.5-25s}$, volume in $T_{max}^{9.5-25s}$ map; NIHSS, National Institutes of Health Stroke Scale at admission. * *p* < 0.05.

The $T_{max,v}^{16-25s}$ value did not significantly differ between the two groups. Box and whisker plots of $T_{max,v}^{16-25s},T_{max,v}^{9.5-25s}$, CBV_v_, CBF_v_, $T_{max,v}^{9.5-25s}$ − CBF_v_, and NIHSS scores are illustrated in Figure 3.

No differences in the rate of collateral flow or collateral flow score was observed (*p* = 0.9).

## 4. Discussion

The aim of this study was to investigate the effect of premorbid statin treatment on CT perfusion findings in patients with acute ischemic stroke due to LVO before reperfusion therapy. Our main results show that premorbid statin use is associated with smaller volumes on several perfusion maps, reflecting lower ischemic core and ischemic penumbra volumes. Nevertheless, no statically significant difference was detected for $T_{max,v}^{16-25s}$, which estimates the volume of the ischemic core. In our opinion, this should not be interpreted as a negative result since it underlines a more prominent role of statins at the level of the ischemic penumbra rather than ischemic core.

Despite the reported effect of statins on cerebrovascular reactivity and endothelial function [7,11,12], we were unable to demonstrate an association with good collateral flow. In our opinion, this could be due to the small sample size but also to the scale used.

Indeed, while the method proposed by Tan et al. and the PROACT II study is easily reproducible, supported by solid evidence, and allows for rapid application (an important aspect in the emergency management of acute ischemic stroke patients), it might not be sensitive enough to detect the effect of statins on leptomeningeal collaterals [30]. However, evidence in the literature suggests otherwise. Several studies have shown that patients with good collateral flow have a smaller infarct core, as assessed using perfusion CT variables, which may have greater sensitivity than methods based solely on CTA.

Several studies have shown that patients with good collateral flow have a smaller infarct core, as assessed using perfusion CT variables [31,32,33]. A study conducted by Zhu and colleagues demonstrated that the utilization of statins prior to the onset of stroke was independently associated with favorable leptomeningeal collaterals, as assessed using CTA with a detailed single-phase CTA score with 20 points [33]. A meta-analysis highlighted that statin therapy was associated with good collateral scores in patients with acute ischemic stroke volume; however, the selected studies assessed collaterals using digital subtraction angiography [6]. Future research should focus on the effect of pretreatment statins using combined approaches for collateral estimation that are ideally automated and with AI support [34]. Evidence on the clinical effect is limited. Xu et al. reported, in their subgroup analyses, that patients with statin use were related to better collateral status on good mRS [35]. Otherwise, Nannoni et al. reported that known or newly diagnosed hypercholesterolemia was associated with better collaterals, suggesting a possible link between hypercholesterolemia and collateral extension likely related to the increased levels of vascular endothelial growth factor [36]. Surely, the effects of statins on endothelial function may reasonably influence CTA and CTP findings. In addition to the known effects on eNOS and neoangiogenesis, a direct antithrombotic effect has been clearly reported in arterial and venous thrombosis models via mechanisms unrelated to cholesterol-lowering activity. Statins may inhibit several pathways of hemostasis, including platelet activation and the coagulation cascade [37,38].

Global hypoperfused volume was the parameter with the largest difference between groups. This is an important finding given that global hypoperfused volume is related to clinical severity and that the NIHSS at baseline was lower in the statin group.

To our knowledge, only one previous study from Kumar et al. evaluated perfusion parameters in acute ischemic stroke according to premorbid therapy [39]. Consistent with this study, we found that the NIHSS at onset and the volume of tissue at risk were reduced in patients with premorbid therapy. However, the study by Kumar et al. differs from the present as the patients were evaluated within 24 h from symptom onset (in less than 70% of patients within 6 h of stroke), and perfusion status was assessed using magnetic resonance imaging. In contrast, our findings relate to the hyperacute phase of ischemic stroke and are based on multimodal CT imaging. This confirms the feasibility of CT for the assessment of ischemic stroke in the hyperacute pretreatment setting, as all patients in our sample were imaged within 6 h of onset. In their study, Kumar et al. found an association with reduced mismatch volume (penumbra-core) only, but not with ischemic core or ischemic penumbra alone [39]. Our study remains the first to investigate the effect of premorbid statin in patients eligible for mechanical thrombectomy.

This study was not designed to evaluate the effect of statins on functional outcome, but it was focused on the very early stage of stroke. Moreover, detailed data on continuation of statin therapy after onset of symptoms were not available. In addition, in the majority of cases, statin treatment was discontinued in the first few days because of dysphagia, which may explain the lack of clinical benefit, as previously reported [5].

There are several limitations in our study. First, this was a non-randomized observational study. Second, the sample size of the two groups of patients was relatively small. However, the patients were selected according to strict criteria and were homogeneous regarding sex, age, and vascular risk factors. Other confounders may be unmeasured factors such as better socioeconomic status, medication adherence, and lifestyle in the statin group, which may have influenced our results [6]. Even though most patients were on statins for primary prevention of hypercholesterolemia using atorvastatin (10–40 mg) and simvastatin (up to 20 mg), data on the effects of statins and dose–response relationships are not available for the entire enrolled population. Furthermore, information on the duration of statin treatment before the acute ischemic event was not available. However, we were able to report on the type and average dose of statins, and some trials clearly showed an early (3–7 days) platelet inhibitory effect after the start of treatment [40].

Another limitation is the lack of follow-up and long-term clinical outcomes after reperfusion treatment.

Referring to the rationale behind the selection of specific cutoff values of CT perfusion maps that may differ from the routinely used software, we do not feel that it is a real limit. We must keep in mind that the perfusion map values are strictly related to the commercial CT perfusion software used, since an intrinsic variability of ischemic lesions assessed by different CTP software packages has been reported [26,27,28,41,42]. Tmax is a perfusion parameter used both in CTP and PWI-MRI and reflects the time delay between the contrast bolus arriving in the proximal large vessel arterial circulation (arterial input function) and the brain parenchyma [43]. Even though a Tmax > 6 s is commonly used to identify globally hypoperfused tissues (penumbra + ischemic core), other colored overlay maps (as Tmax > 10 s and Tmax > 16 s) are however routinely shown to help visualize severity gradations within the hypoperfused areas. A CTP-derived Tmax threshold of around >16 s on average, in both gray and white matter, seems to have the highest sensitivity and specificity for brain tissue that is infarcted even when reperfused early (within 90 min from CTP imaging). So, Tmax > 16 s suggests critical hypoperfusion or nearly complete vascular occlusion, and often this tissue corresponds to area with poor or no collateral flow (ischemic core) as reported in DEFUSE 2. Otherwise, the rationale for using a Tmax > 9.5 s is to achieve greater specificity to identify the critically hypoperfused area since a lower Tmax might capture hypoperfused tissue that could survive even without reperfusion; furthermore, Tmax > 9.5 s might minimize false positive cases (such as late window patients or patients with low NIHSS score). Therefore, we can consider that a Tmax > 6 s has greater sensitivity (standard for identifying penumbra). On the other hand, a Tmax > 9.5 s improves specificity (identify severely compromised tissue), and Tmax > 16 s may be considered as a prognostic marker of likely unsalvageable or infarcted tissue.

CBV is the amount of blood in a specific amount of brain tissue and typically ranges from 2.5 to 5.8 mL per 100 g of brain tissue [44]. According to the abovementioned scientific article, we set our CBV range in the perfusion map between 1 and 8 mL/100 g of brain tissue to identify areas with different CBV values using a color-coded scale.

CBF is the amount of blood flow in specific areas and in the brain tissue. The normal CBF value in humans is about 50 mL per 100 g of tissue per minute, with values that range from 20 mL/(100 g min) in the white matter and 80 mL/(100 g min) in the gray matter [45,46]. A CBF lower than 80 mL/100 g per minute in brain tissue is related to impaired protein synthesis, selective neural loss, and selective gene expression without abnormalities depicted in CT images mainly related to impaired gray matter. Moreover, a CBF < 10–15 mL/100 g per minute is related to irreversible neuronal dysfunction after 30 min, with increased of extra-cellular ions, and ionic edema. Considering this physiological data, we set the lower and upper limits of CBF values of the perfusion map to 8 mL and 80 mL, respectively.

Our results suggest that statin use before acute ischemic stroke is related to a better neuroimaging status before reperfusion treatment, in terms of volume of tissue at risk and volume of irreversibly damaged tissue. These results need to be confirmed in larger and randomized cohorts to strengthen the relevance of statin treatment for primary prevention in high-risk patients.

## Figures and Tables

**Table 1:** Demographics and baseline patient characteristics.

Patient Characteristics	Statins (n = 29)	No Statins (n = 32)	*p*-Value
Mean age [range] (in years)	75.7 [58–87]	72.7 [45–86]	0.5
Sex (male/female)	11/18	11/21	0.6
Risk factors, n (%)			
-Atrial fibrillation	14 (48.3)	9 (29.0)	0.1
-Systemic hypertension	25 (86.2)	23 (74.2)	0.2
-Smoking	10 (34.5)	5 (16.1)	0.1
-Diabetes	12 (41.4)	6 (19.3)	0.1
-Hypercholesterolemia	19 (65.5)	5 (16.1)	<0.001 *
-Transient ischemic attack/ictus	6 (20.7)	5 (16.1)	0.7
-Coronary artery disease	9 (31.0)	4 (12.9)	0.1
Admission NIHSS, median (IQR)	13 [7–16]	17 [15–20]	<0.001 *

n, number; NIHSS, National Institutes of Health Stroke Scale; IQR, interquartile range; * *p*-value < 0.05 was considered statistically significant.

**Table 2:** Perfusion parameters derived from the statin and no statin groups.

Variables [cm^3^]	Statins (n = 29)	No Statins (n = 32)	*p*
$T_{max,v}^{16-25s}$	24.7 [4.23, 33.06]	35.46 [15.71, 54.96]	0.165
$T_{max,v}^{9.5-25s}$	37.59 [16.08, 102.43]	107.00 [75.05, 173.50]	<0.001 *
CBV_v_	3.60 [0.65, 12.40]	11.85 [5.37, 30.56]	0.015 *
CBF_v_	25.6 [7.84, 59.75]	56.84 [31.26, 87.67]	0.037 *
$T_{max,v}^{9.5-25s}$ − CBF_v_	5.63 [−14.75, 27.75]	40.52 [−9.65, 102.47]	0.035 *

Abbreviations: n, number; $T_{max,v}^{16-25s}$, volume in $T_{max}^{16-25s}$ map, T_max_ between 16 s and 25 s was considered the ischemic core;$\;T_{max,v}^{9.5-25s}$, volume in $T_{max}^{9.5-25s}$ map, T_max_ between 9.5 s and 25 s was considered the globally hypoperfused area; CBV_v_, volume in cerebral blood volume (CBV) map; CBF_v_, volume in cerebral blood flow (CBF) map. Data are expressed as median and 95% confidence interval (CI). *p*-value from the Wilcoxon rank-sum test were reported, * *p* < 0.05.

## Data Availability

The data presented in this study are available on request from the corresponding author due to privacy and ethical restrictions.

## Abbreviations

CBF	Cerebral blood flow
CBV	Cerebral blood volume
CT	Computed tomography
CTP	Computed tomography perfusion
eNOS	Endothelial nitric oxide synthase
EVT	Endovascular therapy
LVO	Large vessel occlusion
MCA	Middle cerebral artery
mCTA	Computed tomography angiography
MTT	Mean transit time
NCCT	Axial non-contrast CT
NIHSS	National Institutes of Health Stroke Scale
PROACT	Prolyse in Acute Cerebral Thromboembolism
ROI	Region of interest
